# Cell State Transitions and Phenotypic Heterogeneity in Luminal Breast Cancer Implicating MicroRNAs as Potential Regulators

**DOI:** 10.3390/ijms24043497

**Published:** 2023-02-09

**Authors:** Vinitha Richard, Madhumathy G. Nair, Vishnu S. Jaikumar, Sara Jones, Jyothi S. Prabhu, Michael J. Kerin

**Affiliations:** 1 Discipline of Surgery, Lambe Institute for Translational Research, University of Galway, H91 V4AY Galway, Ireland; 2 Division of Molecular Medicine, St. John’s Research Institute, Bangalore 560034, Karnataka, India; 3 Rajiv Gandhi Centre for Biotechnology, Thiruvananthapuram 695585, Kerala, India

**Keywords:** luminal breast cancer, cancer stem cells, microRNA, tumorigenic potential, hormone receptors, phenotypic transition, tumor subtypes, side population cells, drug transporter proteins, intratumoral heterogeneity

## Abstract

Luminal breast cancer subtypes respond poorly to endocrine and trastuzumab treatments due to cellular heterogeneity arising from the phenotype transitions, accounted for mainly by the loss of receptor expression. The origins of basal-like and human epidermal growth factor receptor 2 (HER2)-overexpressing breast cancer subtypes have been attributed to genetic and protein modifications in stem-like cells and luminal progenitor cell populations, respectively. The post-transcriptional regulation of protein expression is known to be influenced by microRNAs (miRNAs) that are deemed to be master regulators of several biological processes in breast tumorigenesis and progression. Our objective was to identify the fractions of luminal breast cancer cells that share stemness potentials and marker profiles and to elucidate the molecular regulatory mechanism that drives transitions between fractions, leading to receptor discordances. Established breast cancer cell lines of all prominent subtypes were screened for the expression of putative cancer stem cell (CSC) markers and drug transporter proteins using a side population (SP) assay. Flow-cytometry-sorted fractions of luminal cancer cells implanted in immunocompromised mice generated a pre-clinical estrogen receptor alpha (ERα+) animal model with multiple tumorigenic fractions displaying differential expression of drug transporters and hormone receptors. Despite an abundance of estrogen receptor 1 (ESR1) gene transcripts, few fractions transitioned to the triple-negative breast cancer (TNBC) phenotype with a visible loss of ER protein expression and a distinct microRNA expression profile that is reportedly enriched in breast CSCs. The translation of this study has the potential to provide novel therapeutic miRNA-based targets to counter the dreaded subtype transitions and the failure of antihormonal therapies in the luminal breast cancer subtype.

## 1. Introduction

Despite the advancements in early detection techniques and neo-adjuvant or adjuvant therapies promoting a decline in the long-term mortality rate for breast cancer worldwide, it remains the leading cause of death in women aged 20 to 59 years [1,2,3]. Although the normal mammary gland consists of a modest three morphological lineages (the ductal, alveolar, and myoepithelial cells), these epithelial cells transform into functionally complex and molecularly heterogeneous multiple breast tumor subtypes [4,5,6] with a distinct histology, genomic and transcriptomic profiles, and responses to therapy [7]. While there are advanced genomic profiling techniques demonstrating multiple profiles, the scenario of biological heterogeneity is identified in clinical practice by the differential expression of hormone receptors such as estrogen receptor (ER), progesterone receptor (PR), and human epidermal growth factor receptor 2 (HER2) [6]. Of these, (ERα), and ERBB2/HER2 are the two main targetable tumorigenic drivers [8] and the third marker PR (*NR3C3*) is an ERα target gene that indicates active estrogenic signaling and improved prognosis in comparison to PR-negative tumors [9]. Although more than two-thirds of breast cancer cases are ERα+ or PR+, 25% to 40% of patients relapse after one or several lines of antihormonal (endocrine) treatment [10,11,12].

The clinical scenario of tumor relapse and the emergence of aggressive drug-resistant cancer cells may be ascribed to the presence of fractions with self-renewal properties of normal mammary progenitor cells known as cancer stem cells (CSCs) [13,14,15]. The experimental evidence shows that antiestrogen therapies targeting a mixed lineage of ERα+ breast cancers expressing both markers of normal luminal and basal epithelial cells are inadequate in limiting cancer cell proliferation, but are capable of inducing adaptive responses such as tumor cell plasticity that promote the emergence of basal tumor subtype with enhanced CSC self-renewal activity [12,16]. Interestingly, estrogen has no reported role in progenitor or stem cell activity, but it increases the expression of the downstream target PR, which indirectly mediates the expansion of the stem cell population [17]. Despite interventional therapy, there is currently a dearth of knowledge about the causes of this subtype shift, and there are no active targets or leads available to circumvent this luminal to basal cancer cell enrichment.

Tumorigenic breast CSCs have been reportedly enriched in cells with a CD44+/CD24− cell surface phenotype [14,18] and dye-effluxing side population (SP) cells, which express high levels of drug transporter proteins, ATP-binding cassette subfamily-B member 1 (ABCB1)/MDR1/CD243, and ATP-binding cassette subfamily-G member 2 (ABCG2)/BCRP/CD338 [19,20]. Previous studies have reported the presence of stem-like cell-enriched fractions expressing CD147 and multiple drug transporter proteins in oral and breast tumors [19,21]. These subpopulations were perceived to survive by undergoing phenotypic transitions between a hyperplastic drug transporter expression phase and a dormant drug-sensitive phase underpinning the CSC shift hypothesis [22]. We previously demonstrated the co-existence of two additional fractions each of drug transporter protein-expressing (ABCG2+/MDR1+) side population (SP1 and SP2) cells and non-expressing (ABCG2−/MDR1−) main population (MP1 and MP2) fractions (also known as non-side population (non-SP) cells), respectively, which incessantly replenished the other fractions during multiple cell culture passages and vividly reflected the potential for tumor plasticity [19,23]. Studies also prove that CD338+/ABCG2+ cells have an adaptive advantage in vivo over the differentiated bulk of cancer cells [23], and notably both ABCG2+ and ABCG2− cell subpopulations were observed to display similar tumorigenic potential [24]. Through this study, we intend to derive an association between drug transporter proteins as alternatives to stemness markers, the tumor initiation potential of stem-like cancer cells, and the apparent receptor discordance in breast cancer.

Mammary hormones estrogen and progesterone are also known to regulate the self-renewal of CSCs through small non-coding RNAs known as microRNAs that have dual functions as oncogenes and tumor suppressors by repressing the expression of multiple target genes by degrading the target mRNAs or by inhibiting the translation to functional proteins [6,25,26,27]. The differential expression of microRNAs also correlated with the transient phenotypic shift between cancer cell fractions, leading to the emergence of highly tumorigenic stem-like cells among the differentiated, non-tumorigenic bulk of cancer cells [28]. Although multiple miRNAs are modulated by ERα activation, the reverse action of the miRNA-mediated regulation of ERα expression and subsequent downstream oncogenic signaling pathways are reported to be linked to acquired resistance to standard first-line endocrine therapies [29,30].

This study aims to devise a strategy to accurately identify, isolate, and characterize the highly transformative, drug-resistant stem-like populations that display a high expression level of stemness markers and are detectable using the gold standard method of in vivo tumorigenic potential measurement in immunocompromised mice. We also intend to develop a heterogeneous lineage ERα+ preclinical breast tumor animal model and characterize the fractions to determine the molecular regulatory mechanisms governing the phenotypic transitions and aiding in the prediction of acquired resistance to targeted therapy. Through this manuscript, we provide experimental proof that the differential expression of microRNAs might be one of the epigenetic regulatory mechanisms guiding the transitions in fractions enriched for stem and progenitor cell populations.

## 2. Results

### 2.1. Cell Surface Expression of Stem-Cell-Associated Markers in Breast Cancer

**Figure 1:**
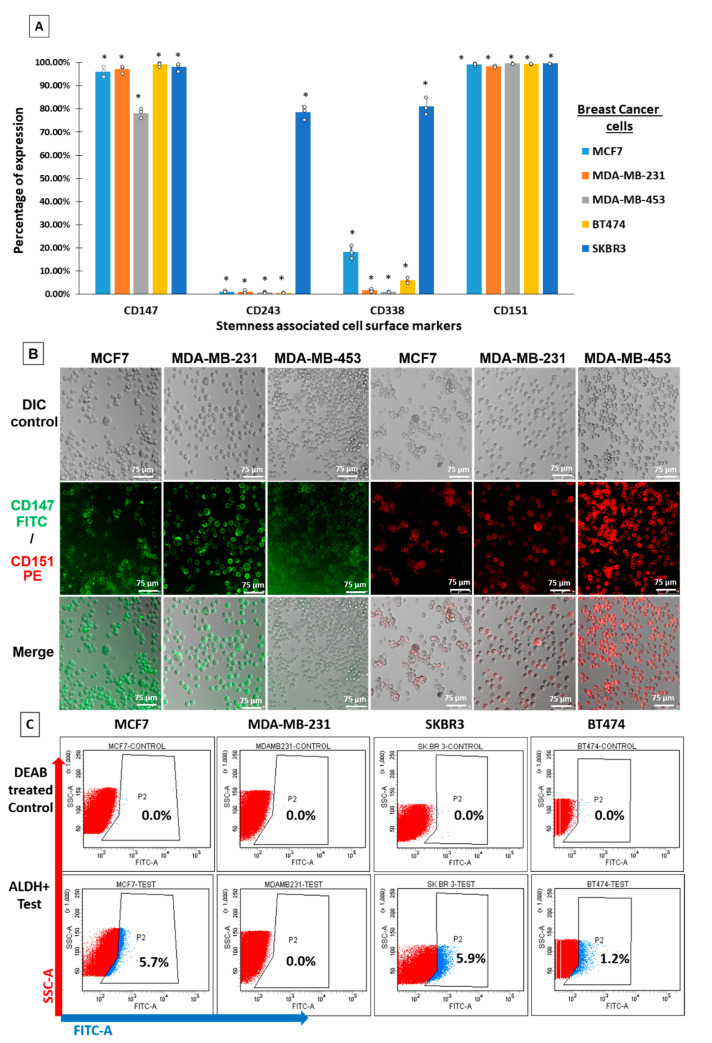
Expression profiling of stemness-associated markers in breast cancer. (**A**) Breast cancer cell lines were screened for the expression of the cell surface marker CD147/Basigin or EMMPRIN, CD151/tetraspanin, and drug transporter proteins CD243/ABCB1 and CD338/ABCG2. More than 5% of MCF7, BT474, and SKBR3 cells expressed drug transporter proteins (* comparison between unstained control and stained cells for each cell line, *p* ≤ 0.05; calculated by Student’s *t*-test). (**B**) Immunofluorescence staining for CD147 and CD151 (scale bar measures 75 µm). (**C**) Breast cancer cell lines MCF7, BT474, and SKBR3 showed positive expression for aldehyde dehydrogenase activity (ALDH), indicating the enrichment of cells with higher self-renewal and drug resistance potential.

The immunophenotyping of different breast cancer cell lines revealed that all cells displayed high expression levels of CD147 and CD151, irrespective of the subtype (Figure 1A), which was also confirmed by confocal imaging (Figure 1B). Nearly 80% of the SKBR3 breast cancer cells were highly positive for the expression of both drug transporter proteins ABCG2/CD338 and MDR1/CD243, while 10% of the MCF7 cells expressed CD338/ABCG2 (Figure 1A). The TNBC cell line MDA-MB-231 displayed the lowest expression level of drug transporters, indicating a subtype-specific and distinctive functional role of these proteins in mediating stemness and aggressiveness in HER2+ and luminal A breast tumor subtypes.

### 2.2. ALDH1-Expressing Stem-like Cells with High Self-Renewal Potential

The screening of breast cancer cell lines for ALDEFLUOR positivity revealed that nearly 1.2–5% of BT474, SKBR3, and MCF7 cells were enriched for ALDH1 activity (Figure 1C). The percentage of cancer cells in MCF7 and SKBR3 cell lines that expressed drug transporter proteins and displayed ALDH1 activity was approximately 5% of the cells, which correlated with the concept of small fractions of CSCs reportedly being enriched in breast cancer cell lines.

### 2.3. Identification of Dye-Effluxing Stem-Cell-Enriched Fractions in Breast Cancer

**Figure 2:**
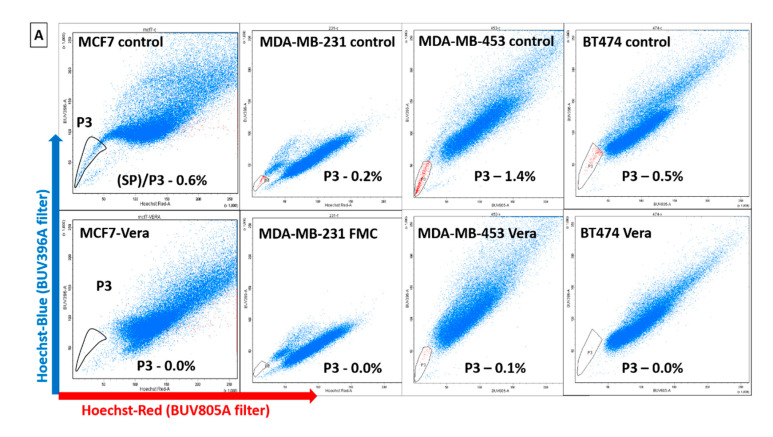

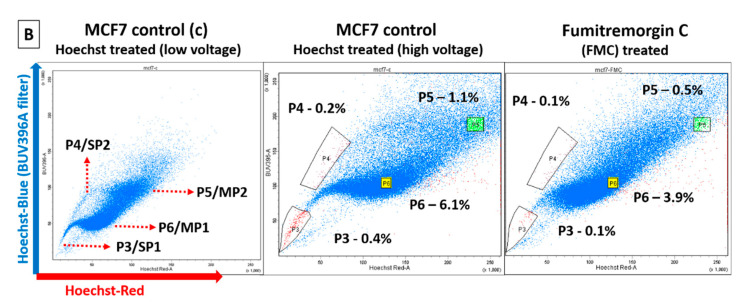
Screening for dye effluxing fractions in breast cancer. (**A**) Hoechst dye efflux activity was displayed by cancer cells across all four major breast cancer subtypes. Hoechst^low^ side population (SP cells gated in P3) and Hoechst^high^ brighter cells retaining the dye were the non-side population cells. The identity of the SP fraction was confirmed via treatment with the drug transporter protein inhibitors verapamil (Vera) and fumitremorgin C (FMC). (**B**) Predominantly, the MCF7 cells displayed a higher percentage of Hoechst^low^ cells as two separate fractions differing in their DNA content (dual side population fractions SP1 (gated in P3) and SP2 (gated in P4)) within the hummingbird pattern and were chosen for additional experiments. Corresponding Hoechst^high^ dye-retaining non-side population cells (MP1 gated in P6 and MP2 gated in P5) were also selected for comparison.

Drug transporter protein-expressing fractions corresponding to SP cells were also observed in breast cancer cells treated with Hoechst 33342 dye (Figure 2A). From analyzing an average of 60,000 live cells (blue cells in the histogram) in the P2 gate, we observed that nearly 0.2–1.4% of cells were positive for the expression of the MDR1 drug transporter protein, as evidenced by the low retention of Hoechst dye. Multiple fractions expressing drug transporter proteins were gated in P3 and P4 of the MCF7 cells and were termed SP1 and SP2 (Figure 2B). The group of cells gated in P5 and P6 termed the main population cells (MP1 and MP2) corresponded to the non-drug transporter-expressing fractions and retained the dye post-treatment with ABCG2 and MDR1 inhibitors FMC and Vera, respectively (Figure 2B). The MP1 and MP2 fractions seemed to be derivatives of SP1 and SP2 cells, but with a higher DNA content and enhanced staining with dye. Since the MCF7 cells displayed a clear resolution of the distinctive dye efflux activity of all four fractions, this cell line was selected as a study model for additional experiments. The sorted cell fractions were re-sorted using the same flow cytometry parameters to ascertain a purity of 100%. The cell viability post-sorting was confirmed by staining with trypan blue (Supplementary Figure S1).

### 2.4. Orthotopic Breast Tumor Regeneration Potential in NSG Mice

**Figure 3:**
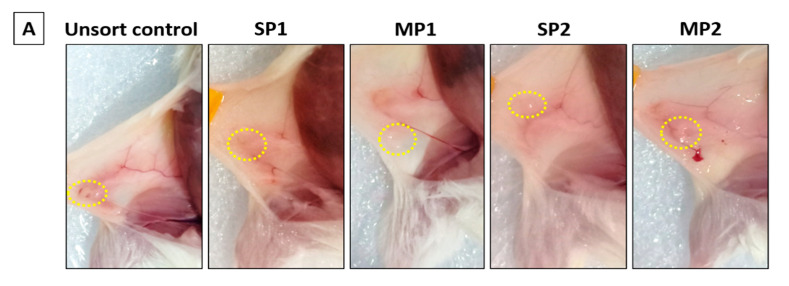

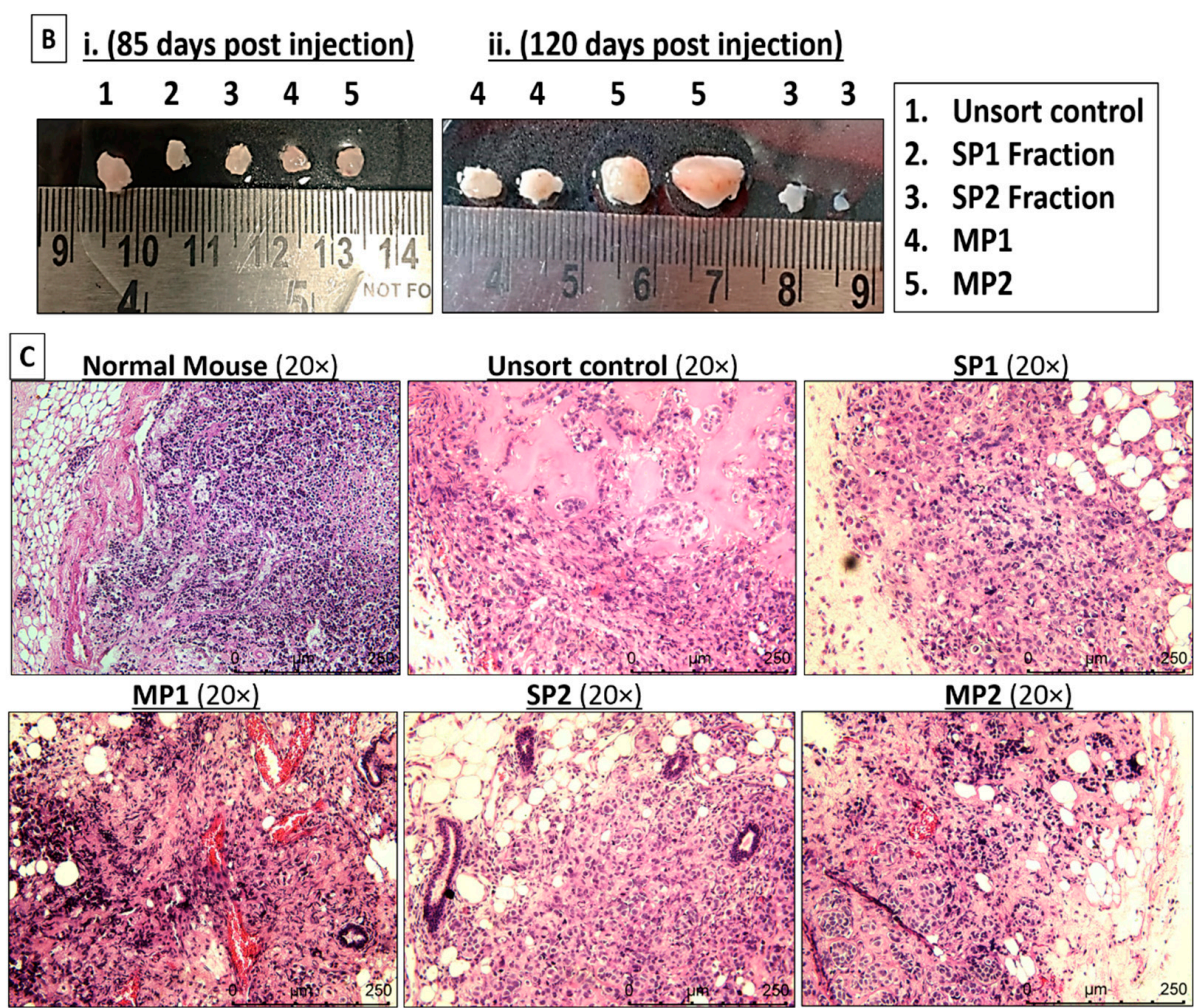
In vivo breast tumor repopulation assay in NSG mice. (**A**) The sorted fractions (SP1, SP2, MP1, and MP2) and a Hoechst-dye-stained unsorted control fraction (UC) of MCF7 cells were injected orthotopically (site of xenograft circled in yellow) in the fat pads of NSG mice (4 mice per fraction) to assess the differential tumorigenic potential of each fraction. (**B**) The xenografts were excised at the time points of (i) 85, 90, and (ii) 120 days after the initial date of injection. There was a visible difference in tumor volume generated by the fractions, indicating a differential rate of cell proliferation over the period of time. (**C**) Hematoxylin and eosin (H&E) staining revealed distinct pathological features such as the tissue morphology, aggressiveness, infiltration of blood vessels, and grade of tumors that varied between the xenograft tumors from UC, SP1, SP2, MP1, and MP2 fractions (scale bar measures 250 µm, representing a magnification of 20×). An image of normal mouse mammary gland tissue was included for comparison between human and mouse breast tissues.

Hoechst-dye-treated MCF7 cells sorted into SP1, SP2, MP1, and MP2 fractions at a final count of 1 × 10^5^ viable cells per fraction were injected orthotopically into the fat pads of NSG mice (4 mice per fraction) to assess the differential tumorigenic potential of each fraction (Figure 3A). Hoechst-dye-treated and unsorted MCF7 cells were injected as unsorted control (UC) fractions to exclude any potential toxic effect of the dye. All sorted fractions were highly tumorigenic, even at low cell counts, and the NSG mice generated palpable orthotopic xenografts within 85 days post-injection. The samples were retained in the animal for 120 days in total before euthanizing the animal (Figure 3B). The xenografts formed post-injection of the unsorted control (UC), MP1, and MP2 fractions displayed angiogenic potential, with a prominent blood vessel originating from the tumor site to the abdomen (Figure 3A). Although the initial transplanted cell load was very small, the tumors generated in 85–90 days were similar in size across all fractions (Figure 3B) but differed post-120 days of transplantation (Figure 3B). The MP2 fraction generated large-sized tumors post-120 days, highlighting an active cell proliferation potential, whereas relatively smaller-sized tumors were generated from MP1 and SP2 and negligible-sized tumors for SP1 fractions, respectively (Figure 3B).

### 2.5. Histopathological Analysis of Xenografts in NSG Mice

The hematoxylin and eosin (H&E) staining of xenograft tumors from UC, SP1, SP2, MP1, and MP2 fractions revealed a distinctive tissue morphology across the fractions (Figure 3C). The tumors from the UC fraction were more hyalinized, with an apparent loss of tissue architecture, and were admixed with mice and cancer cells of human origin (Figure 3C). The tumors from the SP1 fraction resembled grade III tumors with an infiltrating ductal carcinoma (IDC) morphology with multiple ductal elements and were enriched with poorly differentiated cells with an altered nuclear–cytoplasmic ratio. The xenografts from the SP2 fraction were grade II tumors with an abundance of normal mammary ducts retaining the tissue architecture. The tumors from the MP1 fraction were more dysplastic and had elongated cells indicative of an EMT phase. There were more ducts at the periphery of the tissue and multiple infiltrating blood vessels, indicating angiogenesis. The MP2-fraction-generated xenografts, which displayed features of the normal mammary epithelium, were well-differentiated and moderate to mildly dysplastic, with an intact ductal pattern (Figure 3C). The tumors that were maintained in vivo for approximately 120 days were found to be densely packed, forming a glandular node structure, and also recapitulated the above-mentioned tumor morphology (Supplementary Figure S2). The MP2 fraction seemed to contain a highly proliferative group of cells that were well differentiated and the least aggressive, signifying that this fraction was enriched for hyperplastic tumor cells that lacked ABCG2 and MDR1 drug transporter proteins.

### 2.6. Expression Analysis of ER/PR/HER2 Receptor Proteins on Xenograft Tumors

**Figure 4:**
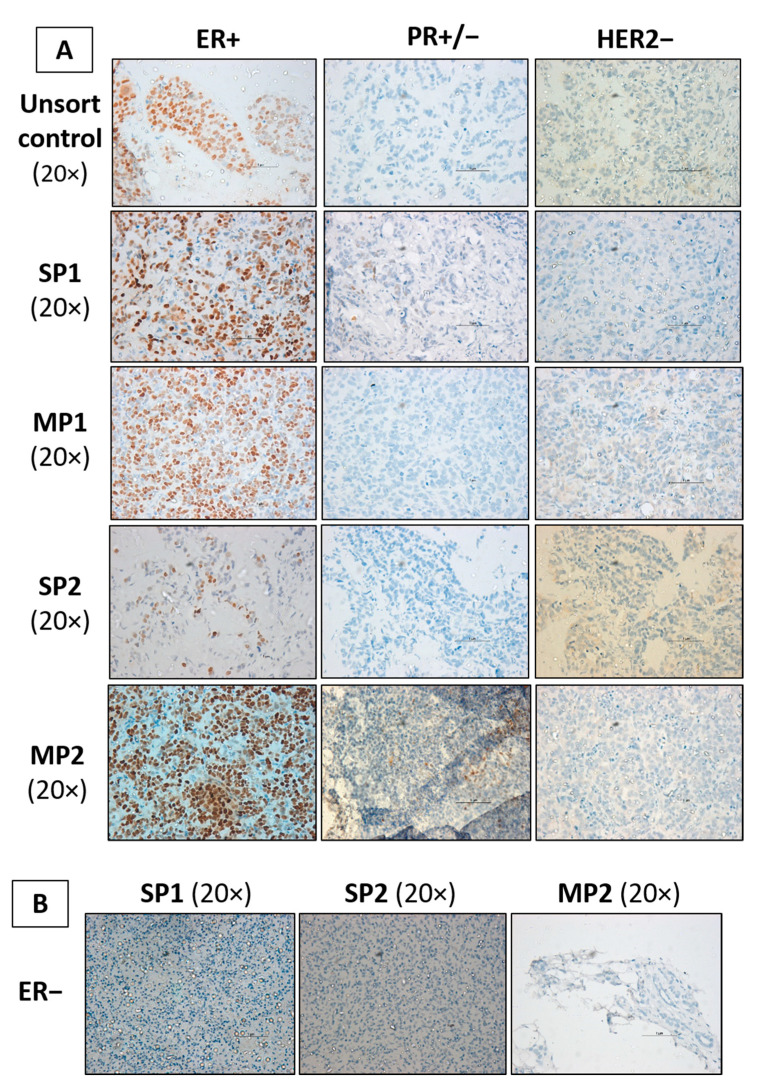
IHC-based expression profiling of hormone receptor proteins. (**A**) The xenografts generated from different fractions in NSG mice were screened for the hormone receptor profile via immunohistochemistry staining. Few samples differed from the parental MCF7 phenotype to develop (ER+/PR−/HER2−) the luminal A subtype with a loss of PR protein expression (PR−). (**B**) The loss of estrogen (ER) and progesterone (PR) receptors in SP1 (85 days), SP2 (120 days), and MP2 (90 days) fractions lead to the (ER−/PR−/HER2−) phenotype. The development of variants across a time period was suggestive of the delayed recurrence and emergence of new subtypes in ER+ breast cancers (scale bar measures 1 µm).

MCF7 cells are reported to display the hormone receptor phenotype (ER+/PR+/HER2−) [31]. To confirm if the cells retained this phenotype post-transplantation, we performed immunohistochemistry (IHC)-based staining for the hormone receptors (Figure 4). Interestingly, each of the quadruplicate xenografts per fraction extracted at different time points showed varying receptor phenotypes demonstrating intratumoral heterogeneity (Table 1). The tumor contents varied from 15–70% in the SP1 fraction to 20–90% in the SP2 fractions, 70–90% in the MP1 fraction, and 70–90% in the MP2 fractions, which also correlated with the tumor sizes of all four fractions (Figure 3B, Supplementary Table S1).

The primary staining of tumor sections was performed with a highly specific antihuman antibody against PR (Dako FLEX monoclonal mouse antihuman PR; Clone PgR 636) that enhanced the sensitivity of the detection, thereby grading the majority of tissue samples as PR-luminal (ER+/PR−/HER2−) and triple-negative (ER−/PR−/HER2−) subtypes, respectively (Figure 4B). The PR-negative samples were cross-verified for PR expression using an alternative highly sensitive antibody (PR Pathnsitu RTU EP2), which confirmed that the SP1, SP2-2, MP1, and MP2-4 samples were positive for the expression of PR and also retained the parental MCF7 phenotype (ER+/PR+) luminal A subtype (refer to Supplementary Table S1). This result suggested that the protein expression may vary based on the antibodies used for the IHC, and alternative molecular biomarkers need to be utilized for the precise subtyping of breast tumors. We also pondered over the question of whether the loss of ER protein expression is reflected at the transcriptomic level or there is a complete loss of the *ESR1* gene.

### 2.7. Scoring for ER Gene Expression (ERness Score) and Tumor Aggressiveness

The gene expression-based molecular subtyping of breast cancers suggested that 88% of the low-ER staining tumors were either categorized as basal-like or HER2-enriched, and only tumors with an ER+ staining intensity of 10% were ideally classified as a luminal subtype of breast cancer [32,33,34]. To facilitate accurate transcript-based scoring, a set of luminal genes that have been reported to highly correlate with the ER-positive phenotype (*ESR1*, *GATA3*, *TFF1*) were selected as the best markers for the prediction of ER status, along with a panel of 3 reference genes (*PUM1*, *RPLP0*, *ACTβ*), and the average expression was depicted as the “ERness (ER gene expression) score” [32,35]. Tumors with ER gene score values above 0.68 were considered to have fully functional ER downstream signaling activity, while tumors with scores of less than 0.68 were classified as the inadequate ER downstream function group [36]. Our experiments demonstrated high ERness scores (~0.99) across all xenograft fractions and control MCF7 cells. This indicated the presence of ER transcripts in cancer cells and fully functional ER downstream signaling activity, and yet the absence of ER protein expression highlighted the interference of post-transcriptional regulation, which prevented the translation of ER gene transcripts to functional ER proteins (Table 2). Herein, we were concerned with the possibility that the receptor-negative cells might arise from cross-contamination with mouse epithelial cells. The discrimination between human and mouse tissues was also achieved using a 40-cycle qPCR assay using mouse-specific primers for housekeeping genes (*RPL13a* and *18s*) and a mouse-specific test gene (*PNMT*). The total cDNA extracted previously from the xenograft samples was used for the assay. The results confirmed that there was no contamination with mouse epithelial cells in our xenografts and our data were valid.

Similarly, HR+HER2− tumors with ER+ scores of 10% and above as assessed by IHC were also reported to have inadequate downstream ER activity, categorizing them as a poor prognostic group endorsing the biological heterogeneity associated with the HR+ breast cancer subtype [32,36]. The use of additional transcript-based scoring using multiple genes that represent survival, proliferation, and apoptotic pathways (aggression score) in comparison to classification using the Ki-67 labeling index led to the identification of *ANLN* (involved in cell growth and migration) and *BCL2* (involved in inhibition of apoptosis) with a significant positive correlation, wherein a high grade was used as the predictor and grade 3 as the determinant. The score was referred to as the tumor aggression probability score, with a cutoff of 0.238 [36]. Our results also proved that all xenograft samples had ERness and aggression scores, culminating in the grouping of all fractions as equally aggressive and tumorigenic (Table 2).

We were intrigued by the causes that led to the subdued expression of ER protein, despite the abundance of gene transcripts. It is widely known that miRNAs can mediate the degradation or translational inhibition of mRNAs by combining with the 3′ noncoding regions of target gene mRNAs, and can negatively regulate the gene expression at the post-transcriptional level, mainly by inhibiting the translation or degradation of targets [6,28]. The classification of samples into comparative groups was performed as mentioned in Table 2 and the distinctive role of miRNAs in the subtype transitions in xenografts was assessed using NanoString nCounter Human v3 miRNA Panel microarray following the manufacturer’s protocol.

### 2.8. Differential Expression of MicroRNAs in Multiple Tumorigenic Fractions

**Figure 5:**
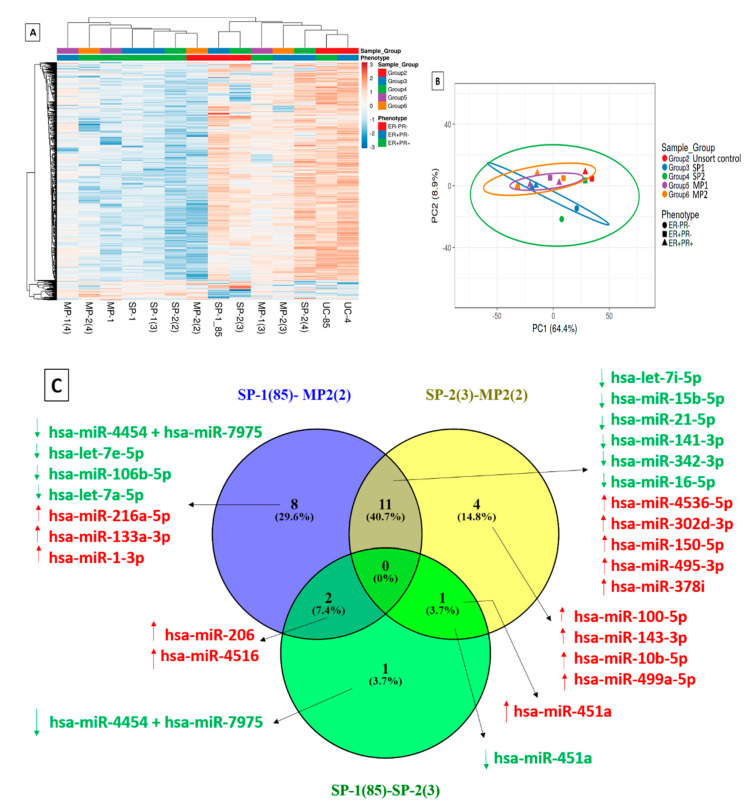
Heat map and PCA mapping of differentially expressed microRNAs. (**A**) MicroRNA array of various subtype phenotypes and a heatmap representation of the differentially expressed (DE) microRNAs. The samples were clustered into two main groups with significantly upregulated and downregulated sets of microRNAs across the fractions. (**B**) The principal component analysis (PCA) plot also grouped the samples broadly into 2 clusters. Samples of the phenotypes ‘ER−PR−’ and ‘ER+ PR−’ were observed to partially cluster together. (**C**) Differential microRNA profiling of ER− fractions in the MCF7 cell line. The differentially expressed miRNAs with significant fold changes ≥ 1.5 (upregulated) or ≤−1.5 (downregulated) and counts ≥ 15.6 (average of negative control) in any of the samples (test or control) are marked as upregulated in red, while the downregulated miRNAs are marked in green. Abbreviations: hsa-miR, *Homo sapiens* (hsa) microRNA.

A heat map was created with the normalized expression of all miRNAs involved in the panel and checked for the similarity of miRNA expression profiles across samples and their downstream clustering (Figure 5A). The principal component analysis (PCA) plot grouped the samples (group 2—unsorted control; group 3—SP1 fractions; group 4—SP2 fractions; group 5—MP1; group 6—MP2 fractions) broadly into 2 clusters. The group 2 unsorted stained control samples were found to be aligned in a single cluster, and samples belonging to different phenotypes were spread all over the heatmap. However, samples of the phenotypes ‘ER−PR−’ and ‘ER+PR−’ were observed to partially cluster together (Figure 5B). Since these were one-to-one sample comparisons that lacked replicates, instead of the *p*-value, the algorithm DE-call in NanoString was implemented to identify whether the difference between the test and control samples was significant or not.

**Figure 6:**
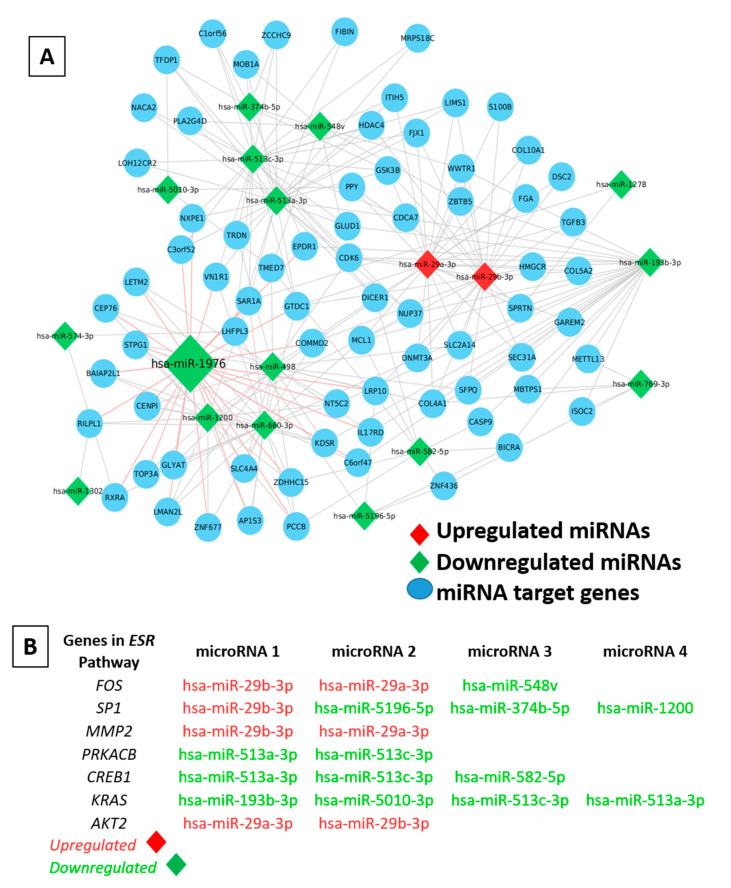
Differentially expressed miRNAs and mRNAs targeting the genes in the ESR pathway. (**A**) A network map of differentially expressed and statistically significant miRNAs across all ER− vs. ER+ samples and the predicted corresponding mRNA targets. The differentially expressed miRNAs with significant fold changes ≥ 1.5 (upregulated) or ≤−1.5 (downregulated) and counts ≥ 15.6 (avg. of negative control) in any of the samples (ER− vs. ER+) are marked as upregulated in red, while the downregulated miRNAs are marked in green. (**B**) *ESR* pathway targeting microRNAs that are differentially expressed between the ER− and ER+ fractions.

The differential expression analysis was performed in 4 different ways. (a) Intergroup molecular variation was analyzed by comparing the miRNA profiles of individual samples from each group against the unsorted or stained control (UC-85), which shared the parental phenotype (ER+PR+) of the MCF7 cells (Supplementary Table S2). In addition, we also conducted an intergroup comparison between groups (3,4,5,6) and a corresponding Venn diagram analysis to identify miRNA patterns distinguishing these subgroups (Supplementary Table S2). Interestingly, it was observed that hsa-miR-195-5p was significantly upregulated in the drug-transporter-expressing SP2 fraction (Group 4) in comparison to the SP1 fraction (group 3). (b) Secondly, an intragroup molecular analysis was performed to determine the miRNA expression patterns of individual samples and to infer how they might have contributed to the phenotypic variation within each group (Supplementary Table S3). No significant differentially regulated miRNAs were detected in the comparison of SP1 (3) vs. SP1, but hsa-miR-126-3p was found to be downregulated by more than 2-fold in both SP-2 (4) vs. SP-2 (2), and SP-2 (2) vs. SP-2 (3) (Supplementary Table S3). (c) In the third analysis, (SP-1 (85), SP-2 (3), and MP-2 (2) samples with a triple-negative phenotype (ER−PR−HER2−) were compared to screen for differences at the molecular level (Figure 5C). (d) In the fourth analysis, the samples were categorized into two major groups, the (i) ER− phenotypic group, and (ii) ER+ group. A comparison was performed between the groups to discern the role of miRNAs in the differential regulation of the ER expression patterns and associated biological functions (Figure 6A). Further, a Venn diagram or overlap analysis was carried out to generate the list of significant differentially expressed miRNAs (Supplementary Table S3). It was observed that one single miRNA (hsa-miR-29a/b-3p) was differentially upregulated in ER− phenotypic fractions and downregulated in ER+ fractions (Figure 6B).

### 2.9. Determining the Role of miRNA–mRNA Interactions in Tumor Plasticity

The mRNA targets were predicted for the differentially expressed miRNAs from the ER− vs. ER+ comparison. A stringent cut-off of an adjusted *p*-value (FDR) ≤ 0.05 or a stringent *p*-value threshold of ≤0.01 was used to reduce the list of mRNA targets. Each statistically significant miRNA from the ER− vs. ER+ analysis was assigned to its corresponding miRNA superfamily, and differentially expressed miRNAs between the ER− and ER+ fractions that target the *ESR* pathway were deduced (Figure 6A). For upregulated miRNAs in each fraction, the corresponding mRNA targets were expected to be downregulated, while for the downregulated miRNAs, the chances were that the pathway remained unperturbed or may have still expressed the mRNA targets. An upregulated expression of hsa-miR-29a/b-3p was observed in ER− fractions of MCF7 cells, which were predicted to target *FOS*, *SP1*, *MMP2*, and *AKT2* genes in the *ESR1*-regulated gene network (Figure 6B).

## 3. Discussion

Cancer cell plasticity is a characteristic related to reversible phenotypic transitions displayed by breast CSCs and by tumor cells undergoing EMT [37]. One of the main features considered to be crucial in the emergence of therapeutic resistance and recurrence of tumor cells is the switch to a different tumor phenotype [38]. Previous studies have shown that a loss of receptors (positive to negative phenotype) is more common than a gain of receptors (negative to positive phenotype), and independent factors such as resistance to endocrine therapy and treatment with trastuzumab have been associated with receptor discordance [38]. Therapy exerts selection pressure on tumor cells, resulting in the enrichment and survival of highly resistant tumorigenic stem-like cells in breast cancer.

The major identification of CSCs and isolation techniques depended on the expression of cell surface markers (CD44+/CD24−/CD147+/CD133+/lin−), ALDH1 enzyme activity, and drug efflux activity of the side population phenotype [15,19,22,39]. The foremost report on the identification of CSCs in breast cancer [13] based on the expression of the antigenic phenotype CD44+/CD24−/EpCAM+/lin− is not a universal representation of tumor-initiating cells (TICs) across all breast cancer subtypes but highlights a heterogeneous mix of normal mammary cells committed to a distinct epithelial–mesenchymal transition (EMT) program [40]. Additional markers such as CD151, CD147, and drug transporter proteins (ABCG2/CD338), (MDR1/ABCB1/CD243) have been reported to interact and co-express with CD44 on cancer cells [19,28,39]. It was also observed that chemotherapy subsequently enriched the fraction of BCSCs, and ALDH1 positivity was significantly more predictive than CD44+/CD24− cell surface markers [41].

ALDH1+ expression is known to elicit the upregulated expression of multiple therapy resistance proteins (p-glycoprotein, GSTpi, or CHK1) [42]. High levels of drug transporter protein expression in BCSCs have been attributed to protecting cells from drug damage via efflux pumping mechanisms [19,20,21,43]. An additional subpopulation of CD44+/CD24+ cells of the BRCA1-mutated basal-A/basal-like breast cancer was sorted using an antibody against CD338 (ABCG2 antigen) and found to be enriched in several stemness markers [23]. It was observed that CD338+ cells were the only cell subpopulation to retain CD326/EpCAM and CD49f/α6-integrin expression, which was also an antigenic phenotype assigned to luminal progenitors [23]. This suggested that the expression of ABCG2/CD338 proteins was specific to the tumor-initiating luminal progenitor subpopulation of BRCA1-mutated breast cancer cells [23].

In this study, we have experimentally proven that there are additional ABCG2/MDR1 drug transporter protein-expressing (SP1 and SP2) fractions in luminal breast cancer that are equally tumorigenic, such as the non-drug transporter-expressing counterparts, the MP1 and MP2 fractions, which are likely to be enriched with the luminal progenitor subpopulation. Although tumor initiation potential was displayed by all fractions injected in NSG mice, even at very low initial cell concentrations of 1 × 10^5^ cells, the size and growth of the xenograft tumors varied with time (85–120 days), as larger tumors were visualized in MP1/MP2 tumors and the tumors generated by SP1/SP2 were comparably reduced in size. The IHC staining for hormone receptors also showed variations in the cell surface expression of hormone receptor proteins (significant losses of either ER, PR, or both) across all fractions. The complete loss of both ER and PR led to the generation of a triple-negative breast cancer (TNBC) phenotype in fractions originating from SP1, SP2, and MP2. This loss and subsequent display of biological heterogeneity in vivo recapitulated the phenomenon of the abrupt emergence of subtype variants in patients subjected to therapeutic interventions such as endocrine therapy, representing a novel means to investigate the molecular mechanisms and potential therapeutic interventions.

The quantitative real-time polymerase chain reaction (qRT-PCR)-derived “ER gene (ER-ness) score” for the expression of the *ESR1* gene and downstream gene targets (*ESR1, GATA3, TFF1*) was confirmed to be an accurate predictor of the ER status in ER low-status tumor samples [32,35]. This scoring effectively differentiated the ER+ >10% from the ER+ ≤ 1–10% samples, which would be ideally categorized as basal or HER2-enriched. An additional scoring (aggression score) system based on the expression of the *ANLN* and *BCL2* genes correlated the ERness score with the tumor grade to predict the intrinsic aggressiveness of the tumors. All tumorigenic fractions from the MCF7 parental cell line were homogenous for the ERness score, indicating uniformity in *ESR1* gene expression. Remarkably, the subsequent variation in the translation of these abundant *ESR1* gene transcripts to ER proteins was indicative of post-transcriptional repression, wherein destabilization of the target mRNAs was found to be the predominant reason for a reduced protein output [6,15,28,44].

MicroRNAs (miRNAs) are small, endogenous ~22-nucleotide RNAs reported to arbitrate important gene-regulatory events by pairing to the multiple mRNA transcripts of protein-coding genes (targets) and directing the repression by decreasing the translational efficiency or by decreasing the mRNA levels, which accounted for most (≥ 84%) of the decreased protein production [6,28,44]. In this study, an miRNA array performed on representative fractions revealed a distinct expression pattern that efficiently categorized the samples into two main clusters and subclusters, even though several of them shared the parental MCF7 cell line phenotype (ER+PR+HER2−). This also highlighted the downside of depending only on hormone receptor profiles for tumor subtyping, clinical decision-making, and predicting the treatment outcome.

The differential miRNA expression profiles of ER+ fractions against ER− samples led to the identification of miRNAs that actively targeted genes in the *ESR* pathway. Our group had previously reported that mir-195 was a circulating biomarker in breast cancer [45] known to act as a tumor suppressor that targets Bcl-2 transcripts in HR+ breast cancers, actively regulating cell proliferation and the cell cycle and also aiding in the suppression of tumorigenicity [46,47,48]. This very much corroborated our finding that orthotopic tumors generated from SP2 and SP1 fractions were smaller in size with limited proliferation potential but were more highly aggressive than MP1/MP2 fractions, further supporting the observation that microRNAs might be responsible for these intrinsic subtype variations.

A recent study provided evidential support that correlated with our results on hsa-miR-29a/b-3p, which was significantly upregulated in the ER- fractions of MCF7 cells and also in CD44+/CD24− breast cancer stem cells (BCSCs) [49]. The microRNA-29b-3p also acted as a promoter in the development of an aggressive TNBC (ER−PR−) cell line MDA-MB-231 [50] and stimulated BCSC migration and invasion both in vitro and in vivo by directly targeting a tumor suppressor gene, *SUV420H2* [49]. Interestingly, CDK6, another target of the hsa-miR-29a miRNA, was found to mediate the recruitment of the transcription factors c-Jun and Sp1 to the MMP2 promoter that positively enriched the angiogenic and fibrotic tumor microenvironment, as widely observed in TNBC (ER−) patient tissues [51]. This in turn reiterated our histopathological results of ER+ xenografts from the MP1 fraction, which was found to be highly infiltrated with lymph and blood vessels, whereas the MP2, SP2, and, SP1 fractions that shared the same ER− phenotype displayed low angiogenic activity.

Additional data supported the findings that the miR-29a-SUV420H2 axis exerted a significant effect on EMT, with subsequent migration and invasion in vitro and eventual tumor dissemination in vivo. Furthermore, hsa-miR-29b-3p was also found to target *TRAF3* and activate the *NF-κB* signaling pathway, while the inhibition of miR-29b-3p significantly reduced the cell viability, cell migration, and invasion by destroying the cytoskeletal integrity [50]. Let-7 and miR-29a were also reported to be higher in ERα− breast cancer cells and known to collectively target and repress Dicer1, thereby leading to a loss of differentiation and an increase in aggressive behavior similar to the TNBC subtype [52]. The collective regulatory mechanisms of these microRNAs potentially influence the ERα status, Dicer protein levels, EGFR, and IGF1R growth factor receptor expression [52]. In summary, the upregulated expression of hsa-miR-29a/b in ER−PR−HER2− fractions (SP1, SP2, and MP2) of the MCF7 cell line has the potential to transform these fractions into a more malignant version enriched with breast cancer stem cells.

## 4. Materials and Methods

### 4.1. Breast Cancer Cell Lines and Monolayer Culture

Breast cancer cell lines of different subtypes utilized for this study are as follows: (i) MCF7 (luminal A, ER+PR+HER2−), (ii) MDA-MB-231 (TNBC, ER−PR−HER2−) were obtained from the American Type Culture Collection (ATCC), Manassas, VA, USA; while (iii) MDA-MB-453 (AR+ER−PR−HER2+FGF+), (iv) BT474 (HER2-enriched, ER−PR−HER2+), and (v) SKBR3 (HER2-enriched) were obtained from M.G.N., SJRI, India [53]. The phenotypic characterization of cell lines was performed as reported previously [31]. The MCF7, BT474, MDA-MB-453, and SKBR3 cells were maintained in the Dulbecco’s modified Eagle’s medium (DMEM, Sigma-Aldrich, St. Louis, MO, USA) containing 25 mM of HEPES and 3.6 g/L of sodium bicarbonate supplemented with 10% fetal bovine serum (FBS, Sigma-Aldrich) and 1% antibiotic antimycotic cocktail (Invitrogen, Carlsbad, CA, USA). The MDA-MB-231 cells were cultured in an L-15 medium (Sigma-Aldrich). All cells were maintained in humidified incubators at 37 °C with 5% CO_2_, and functional cells up to the 10th passage were used for all experiments in this study.

### 4.2. Flow-Cytometry-Based Immunophenotypic Profiling for Stemness Markers

The panel of antibodies used for phenotypic characterization were CD147-FITC (Clone REA282), CD151-PE (Clone REA265), CD243/ABCB1-Viobright FITC (Clone REA495), and CD338/ABCG2-PerCP_Vio700 (Clone 5D3), purchased from Miltenyi Biotec, Gaithersburg, MD, USA). The staining was performed according to the manufacturer’s protocol. Unstained cells were used as negative control. Propidium iodide (PI, Sigma-Aldrich) at a final concentration of (1 μg/mL) was added immediately before analysis to label dead cells, which were excluded from the analysis. The sorting was performed on a fluorescence-activated cell sorter (FACS) Aria III (Becton Dickinson, Franklin Lakes, NJ, USA) and the analysis was performed using Diva software (BD FACSDiva™ 6.0). Additionally, the immunofluorescence cell surface staining was also confirmed by imaging the cells at 40× magnification using a laser confocal microscope (Olympus FV3000).

### 4.3. ALDEFLUOR Assay for Aldehyde Dehydrogenase (ALDH) Enzyme Activity

An ALDEFLUOR™ kit (STEMCELL Technologies, Vancouver, BC, Canada) was used to analyze the level of the enzyme aldehyde dehydrogenase (ALDH) according to the manufacturer’s protocol [17]. Breast cancer cells were trypsinized, resuspended in ALDEFLUOR™ assay buffer, and incubated with the activated ALDEFLUOR™ fluorescent reagent for 30 min (min) at 37 °C. DEAB (*N*,*N*-diethylaminobenzaldehyde), an inhibitor that inhibits the conversion of a substrate to the fluorescent product was added along with the fluorescent reagent in the negative control with gate P2 set at a null value. The cells were resuspended in an assay buffer after incubation and fluorescence signals were detected at 515–545 nm using FACS Aria III (BD Biosciences).

### 4.4. Side Population Assay to Identify the Hoechst33342 Dye Effluxing Fractions

The drug efflux activity of breast tumor cells was analyzed as a side population (SP) assay based on the previously described method [17,19]. The control cells stained with 5 μg/mL of Hoechst 33342 (Sigma-Aldrich) were incubated either alone or in the presence of 75 μM of verapamil (Sigma-Aldrich) and 4 μM of fumitremorgin C (FMC) (Sigma-Aldrich). Propidium iodide (1 μg/mL) was added immediately before the analysis to label the dead cells, which were excluded from further analyses. The flow cytometry sorting of cells was performed in FACS Aria III (BD Biosciences) and the analysis was performed using Diva software. The Hoechst 33342 dye was excited with a UV laser at 355 nm and the emission was collected using 450/50 nm and 396/805 LP filters that allowed the detection of Hoechst blue and red, respectively [17]. The sorted cells in each gated group were collected and re-sorted to ensure a high sort purity. A viability check was performed via staining with trypan blue (Sigma-Aldrich) and the live sorted cells were utilized immediately for animal experiments.

### 4.5. In Vivo Tumorigenic Assay in NOD/SCID Gamma (NSG) Mice

All animal experiments were performed according to a protocol approved by the committee for control and supervision of experiments on animals (CPCSEA) (Reg. No: 326/CPCSEA) and the Institutional Animal Ethics Committee (IAEC) of RGCB (Reg. No. IAEC/618). An approximate 1 × 10^5^ volume of sorted cells of SP1, SP2, MP1, and, MP2 fractions collected in culture medium was resuspended in 100 μL of DPBS mixed with 100 μL of phenol-free, growth-factor-reduced Matrigel (BD Biosciences; 356231). Each fraction was subcutaneously injected in one of the 4th or 9th abdominal mammary glands of 6–7-week-old female NOD.CB17-Prkdcscid/J mice (The Jackson Laboratory, ME, USA) in groups of 4 animals per fraction. As a control for the enrichment of tumor-initiating cells (T-ICs) in sorted fractions, a Hoechst-dye-treated and unsorted whole-cell population was injected at dilutions of 1 × 10^5^ into the NOD/SCID mice. The tumor growth was followed up weekly by palpation and monitored until a size range of 1.0–1.5 cm was reached or the health of the mouse rapidly deteriorated. Upon termination of the experiments, the animals were killed as per CPCSEA guidelines, checked for tumors or metastases (white spots on organs), and tumors were immediately excised and processed for immunohistochemistry.

### 4.6. Immunohistochemistry Staining for Hormone Receptors on Xenograft Tumors

Briefly, formalin-fixed paraffin-embedded (FFPE) breast tumor sections (5 µm in thickness) were cut onto poly L-lysine (PLL)-coated slides, subjected to deparaffinization in xylene, and rehydrated in graded alcohol. After blocking endogenous peroxidase with a 3% hydrogen peroxide (H_2_O_2_) solution, antigen retrieval was performed in 0.01 M EDTA buffer at pH 8 using a heat-triggered MERS (multi-epitope retrieval system; PathnSitu, Pleasanton, CA, USA) for 15 min at 90 °C. The primary blocking was performed with 1% bovine serum albumin (BSA, Sigma-Aldrich) for 30 min at room temperature. The antibodies used for IHC were Dako polyclonal rabbit antihuman c-erbB-2 oncoprotein (1:800 dilution), Dako FLEX monoclonal rabbit antihuman estrogen receptor α, Clone EP1 (Dako Autostainer/Autostainer Plus), Dako FLEX monoclonal mouse antihuman progesterone receptor; Clone PgR 636 (a highly specific antibody ready-to-use, Dako Autostainer/Autostainer Plus; if stained negative, then a second PR antibody that is more sensitive PR Pathnsitu RTU EP2–ready to use was used). The sections were further incubated with the secondary antibody (DAKO REALTM EnVisionTM, Santa Clara, CA, USA) for 20 min as per the kit instructions, followed by the development of the color using DAB (DAKO REALTM EnVisionTM) for 10 min. The sections were counterstained with hematoxylin and mounted after dehydration in graded alcohol and xylene. Appropriate positive and negative controls were run for each batch. The staining patterns were evaluated by two pathologists (J.S.P and S.P.). H&E images were taken in a Leica DM1000 system using a Leica DFC490 camera at 10× and 40× magnifications.

### 4.7. RNA Preparation and Derivation of ERness Score and Aggression Score

Two 20 µm thick FFPE sections from each tumor sample were deparaffinized at 90 °C for 10 min and subjected to overnight digestion at 65 °C with a Tris-EDTA (TE) buffer solution (pH 8.0) containing 1% SDS. The total RNA was then extracted using TRI Reagent (Sigma-Aldrich) according to the manufacturer’s instructions. The quantitation of the RNA was performed using a Qubit™ RNA high-sensitivity (HS) kit. A total RNA concentration of 500 ng was then reverse-transcribed into the cDNA using the ABI high-capacity cDNA archive kit (ABI) as per the manufacturer’s protocol. The transcript levels were measured by qRT-PCR assay in duplicate with a 5 ng template for FFPE sections per reaction, using SYBR Green master mix (SYBRR Premix Ex TaqTM II Tli RNaseH Plus; Takara Bio Inc. Kusatsu, Shiga, Japan) on the Light Cycler 480 II (Roche Diagnostics, Basel, Switzerland) system with a 3 pM concentration of forward and reverse primers made to a final reaction volume of 10 μL. The cycle threshold (CT) values for the test genes (*ANLN*, *BCL2*, *ESR1*, *GATA3*, and *TFF1*) were in turn normalized to the mean CT value of the three reference genes (*ACTB*, *RPLP0*, and *PUM1*) for each tumor sample, designated as the delta CT (∆CT). The relative expression of the test genes was calculated using the ∆CT method as described previously [54]. The ERness score was calculated by fitting a binomial logistic regression model using 3 genes, *ESR1*, *TFF1*, and *GATA3*, and the ER status via IHC (>1%) as the determinant, as published previously [32]. The aggression score was calculated by fitting a binomial logistic regression model using 2 genes, *ANLN* and *BCL2*, as the predictors and grade 3 as the determinant, as published previously [36].

### 4.8. NanoString miRNA Expression Array

The RNA samples were quantified using a Qubit microRNA assay (Invitrogen, Cat #Q32880) kit to assess the miRNA concentration and also qualitatively analyzed on an Agilent 2100 bioanalyzer Pico chip (Agilent technologies, Santa Clara, CA, USA, Cat # 5067-1513). For the NanoString assay, a maximum volume of RNA (3 µL) or 100 ng of miRNA as the input was taken, and the assay was performed according to the manufacturer’s instructions. The analysis was performed on all samples using the nCounter Analysis System (NanoString Technologies, Seattle, WA, USA) and the nCounter Human v3 miRNA Panel containing 799 unique clinically relevant miRNA barcodes along with appropriate positive and negative controls. The raw miRNA data in .RCC (reporter code count) format was further analyzed using the nSolver analysis software version 4.0 (NanoString Technologies). The normalization of the raw data was performed using the geometric mean of the positive controls and the top 100 highly expressed miRNAs as per the instructions in the manual (nCounter Data Analysis Guidelines for miRNA (LBL-C0046-01), nSolverTM 4.0 Analysis Software User Manual (MAN-C0019-08)). The differential expression between the groups was calculated using the build ratio utility (fold change) present within nSolver. The thresholds considered for significant differentially expressed miRNA were a fold change ≥1.5 or ≤−1.5, *p*-value ≤ 0.05 (or DE call predicted ‘YES’), and either of the two groups (test/control) with their geometric mean expression count ≥ 15.6 (avg. of negative control) in any of the samples (test or control). The ClustVis tool (http://biit.cs.ut.ee/clustvis/, (accessed on 20 June 2022) was used for the principal component analysis (PCA) and heatmap generation using the normalized expression data from the samples used in the study. The InteractiVenn web server (http://www.interactivenn.net/, (accessed on 20 June 2022) was used to generate the Venn diagrams.

### 4.9. Target Prediction, miRNA–mRNA Network Mapping, and Functional Analysis

All significant differentially expressed (DE) miRNAs were enriched using the MicroRNA Enrichment Turned Network (MIENTURNET) online tool (http://userver.bio.uniroma1.it/apps/mienturnet/, (accessed on 22 June 2022) by querying them against the miRTarBase database. The thresholds used during the target enrichment were a minimum interaction threshold set at 2 and an adjusted *p*-value (FDR) of 0.05 (or a *p*-value < 0.05 in cases where an FDR < 0.05 was inapplicable). The miRNA–mRNA interaction network was generated using the Cytoscape v.3.7.2 software. The node shapes represented either the miRNA (diamond) or mRNA target (ellipse), while the color of the nodes (miRNA) indicated upregulation (red) and downregulation (green). The functional enrichment analysis was performed using the miEAA 2.0 (https://ccb-compute2.cs.uni-saarland.de/mieaa2/, (accessed on 22 June 2022) online server using the list of significant differentially regulated miRNAs as the input. For the options set during the querying against the KEGG database for pathway enrichment, the *p*-value adjustment method was set to a minimum of 2 hits per subcategory, and the significance level was set to a 0.05 FDR (Benjamini-Hochberg) adjustment.

### 4.10. Statistical Analysis

For the in vitro graphical representations, the results are depicted as the mean ± standard error of mean or standard deviation calculated from two or more experiments. The statistical analysis was performed using Student’s *t*-test in Microsoft excel software version 2016. For all tests, *p* ≤ 0.05 was considered to be statistically significant.

## 5. Conclusions

Estrogen and progesterone receptors have long-standing roles as indicators and predictors of the clinical diagnosis and prognosis of breast cancer. Given the inherent heterogeneity of ER+ breast cancer, the relapse to the ER− tumor subtype post-endocrine therapy, and the enumeration of CSCs in driving tumor progression and disease resistance, there is less evidence connecting the influence of hormone receptors and CSC-mediated cell state transitions. Individualized therapy has now been proven difficult without reliable and predictive biomarkers. We conclude that in addition to screening for the co-expression of stemness-associated markers such as CD147, CD151, CD44, and CD24, drug transporter proteins, namely ABCB1, ABCC1, and ABCG2, also play a major role in aiding the preliminary screening for CSCs that double as metastatic seeds at a later stage. The differential expression of these drug transporter proteins could be identified either through direct targeting by specific antibodies or using a Hoechst dye efflux side population assay. We have successfully identified the presence of multiple tumorigenic fractions sporting the presence and absence of drug transporter proteins (SP1-SP2/MP1-MP2), which serve as ideal in vitro models to study tumor subtype transitions and plasticity. We have also succeeded in validating the CSC shift hypothesis by providing evidence of multiple cell fractions with distinct molecular profiles and outcomes, co-existing in a seemingly homogenous population of breast tumor cells. This would also enable the design of strategies to specifically target all possible combinations of subtype variants that could arise from CSC-enriched aggressive and resistant breast cancer.

Through this study, we have attempted to scientifically explain the reasons for the generation of intra-tumoral heterogeneity in ERα expression and the molecular profile of ER− cells in heterogeneous ER+ tumors. Herein, we provide evidence that the hormone receptor phenotypes may vary and are not conclusive for predicting the aggressiveness of ER+ and ER− subtypes or the outcome of the disease. We also provide details of a miRNA-mediated epigenetic regulatory mechanism that controls the ERα expression and subsequent alteration during breast tumorigenesis, which needs to be investigated further. Silencing the expression of specific miRNAs guiding the phenotypic transitions in combination with conventional therapies might offer an effective mode of preventing subtype transitions, thereby sensitizing tumor cells to therapy. In the near future, we also intend to study the impacts of endocrine therapies and design strategies to impede therapy-induced resistance.

The major limitation of this study was that it focused on a single cell line, MCF7, as an in vitro model of luminal subtypes to address heterogeneity and chemoresistance. An addition of animals per group would have enhanced the statistical significance of the in vivo tumorigenic assays. Additionally, there might be other epigenetic regulatory mechanisms other than miRNAs that are involved in the gain and loss of hormone receptor proteins. The functional validation and silencing of the significantly expressed miRNAs need to be performed for the accurate characterization of the miRNA–mRNA interactome.

## Figures and Tables

**Table 1:** Multiple breast tumor variants in xenografts generated from sorted fractions of MCF7 cell lines. Abbreviations: LVI—lymphovascular invasion; ER—estrogen receptor; PR—progesterone receptor; HER2—human epidermal growth factor receptor; UC—unsorted control; SP—side population; MP—main population.

	No	Sample IDs	Phenotype	Tumor Content	Grade	ER	PR	HER2	Subtype	LVI
Group 1	1	MCF7 cells (+control)	ER+PR+	P7-15	--	+	+	−	ER+PR+HER2−	−
Group 2	2	UC-85	ER+PR+	90%	3	+	+	−	ER+PR+HER2−	−
	3	UC(4)- 120 days	ER+PR−	60%	2	+	−	−	ER+PR−HER2−	−
Group 3	4	SP-1 85	ER−PR−	15%	2	−	−	−	ER−PR−HER2−	−
	5	SP-1-90 days	ER+PR+	35%	2	+	+	−	ER+PR+HER2−	−
	6	SP-1 (3)- 120 days	ER+PR+	70%	3	+	+	−	ER+PR+HER2−	−
Group 4	7	SP-2 (2)- 90 days	ER+PR+	90%	2	+	+	−	ER+PR+HER2−	−
	8	SP-2 (3)- 120 days	ER−PR−	20%	2	−	−	−	ER−PR−HER2−	−
	9	SP-2 (4)- 120 days	ER+PR−	35%	2	+	−	−	ER+PR−HER2−	−
Group 5	10	MP-1- 85 days	ER+PR+	70%	2	+	+	−	ER+PR+HER2−	−
	11	MP-1 (3)- 120 days	ER+PR+	80%	3	+	+	−	ER+PR+HER2−	−
	12	MP-1 (4)- 120 days	ER+PR−	90%	3	+	−	−	ER+PR−HER2−	−
Group 6	13	MP-2 (2)- 90 days	ER−PR−	70%	2	−	−	−	ER−PR−HER2−	−
	14	MP-2 (3)- 120 days	ER+PR−	85%	3	+	−	−	ER+PR−HER2−	−
	15	MP-2 (4)- 120 days	ER+PR+	90%	3	+	+	−	ER+PR+HER2−	−

**Table 2:** Correlation of ER-IHC expression and ER transcript scores. Abbreviations: *ANLN*—anillin; *BCL2*—B-cell lymphoma 2; *ESR1*—estrogen receptor 1; *GATA3*—GATA-binding protein 3; *TFF1*—trefoil factor 1. [ER score cut-off = 0.68; aggression score cut-off = 0.24].

	No	Sample	Protein Phenotype	ERness Score (*ESR1-GATA-TFF1*)	Aggression Score (*ANLN-BCL2*)
Group 1	1	MCF7	ER+PR+HER2−	0.997853409	0.805446153
Group 2	2	UC-85	ER+PR+HER2−	0.996277819	0.541930166
	3	UC-4	ER+PR−HER2−	0.990851261	0.51834784
Group 3	4	SP1-85	ER−PR−HER2−	0.999199652	0.575028151
	5	SP1	ER+PR+HER2−	0.995963034	0.820470204
	6	SP1-3	ER+PR+HER2−	0.999009728	0.723504152
Group 4	7	SP2-2	ER+PR+HER2−	0.998176405	0.785293087
	8	SP2-3	ER−PR−HER2−	0.99677685	0.463903111
Group 5	9	MP-1	ER+PR+HER2−	0.992496709	0.580040679
	10	MP1-3	ER+PR+HER2−	0.996628041	0.774492501
	11	MP1-4	ER+PR−HER2−	0.99793024	0.863435597
Group 6	12	MP2-2	ER−PR−HER2−	0.998328341	0.726683827
	13	MP2-3	ER+PR−HER2−	0.995958468	0.786116171
	14	MP2-4	ER+PR+HER2−	0.998531337	0.502156808

## Data Availability

All data generated during this study are included in this article.

## Supplementary Materials

The following supporting information can be downloaded at: https://www.mdpi.com/article/10.3390/ijms24043497/s1.

## Abbreviations

BC—breast cancer; RNA—ribonucleic acid; miRNA—micro ribonucleic acids; mRNA—messenger ribonucleic acid; HER2—human epidermal growth factor receptor 2; SCs—stem-like cells; CSC—cancer stem cell; SP—side population; NSG—NOD/SCID gamma; ER—estrogen receptor; PR—progesterone receptor; ESR1—estrogen receptor 1 gene; TNBC—triple-negative breast cancer; ABCB1—ATP-binding cassette subfamily-B member 1; ABCG2—ATP-binding cassette subfamily-G member 2; MP—main population; non-SP—non-side population; ATCC—American type culture collection; DMEM—Dulbecco’s modified Eagle’s medium; FBS—fetal bovine serum; PBS—phosphate-buffered saline; BSA—bovine serum albumin; PI—propidium iodide; ALDH—aldehyde dehydrogenase; DEAB—*N*,*N*-diethylaminobenzaldehyde; FACS—fluorescence-activated cell sorter; FMC—fumitremorgin C; CPCSEA—Committee for Control and Supervision of Experiments on Animals; IAEC—Institutional Animal Ethics Committee; T-ICs—tumor-initiating cells; FFPE—formalin-fixed paraffin-embedded; IHC—immunohistochemistry; CT—cycle threshold (CT) values; EMT—epithelial–mesenchymal transition.
