# Supplementary figures and images for: Cell State Transitions and Phenotypic Heterogeneity in Luminal Breast Cancer Implicating MicroRNAs as Potential Regulators
Source: Int J Mol Sci. 2023 Feb 9;24(4):3497. doi: 10.3390/ijms24043497 (PMC9967449; doi:10.3390/ijms24043497)

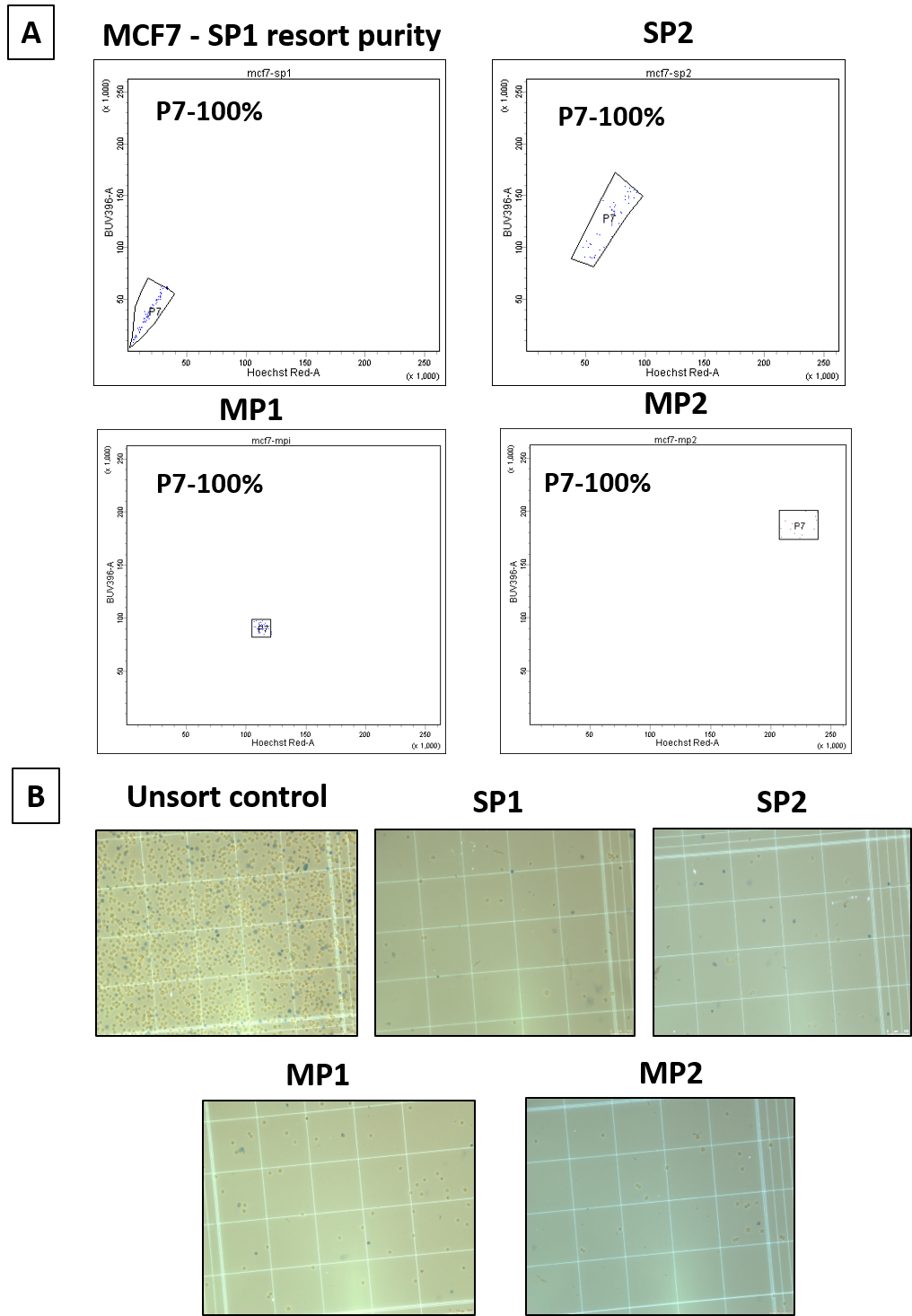

Supplement: Supplementary file 1 [file ijms-24-03497-s001.zip › Figure S1.tif]

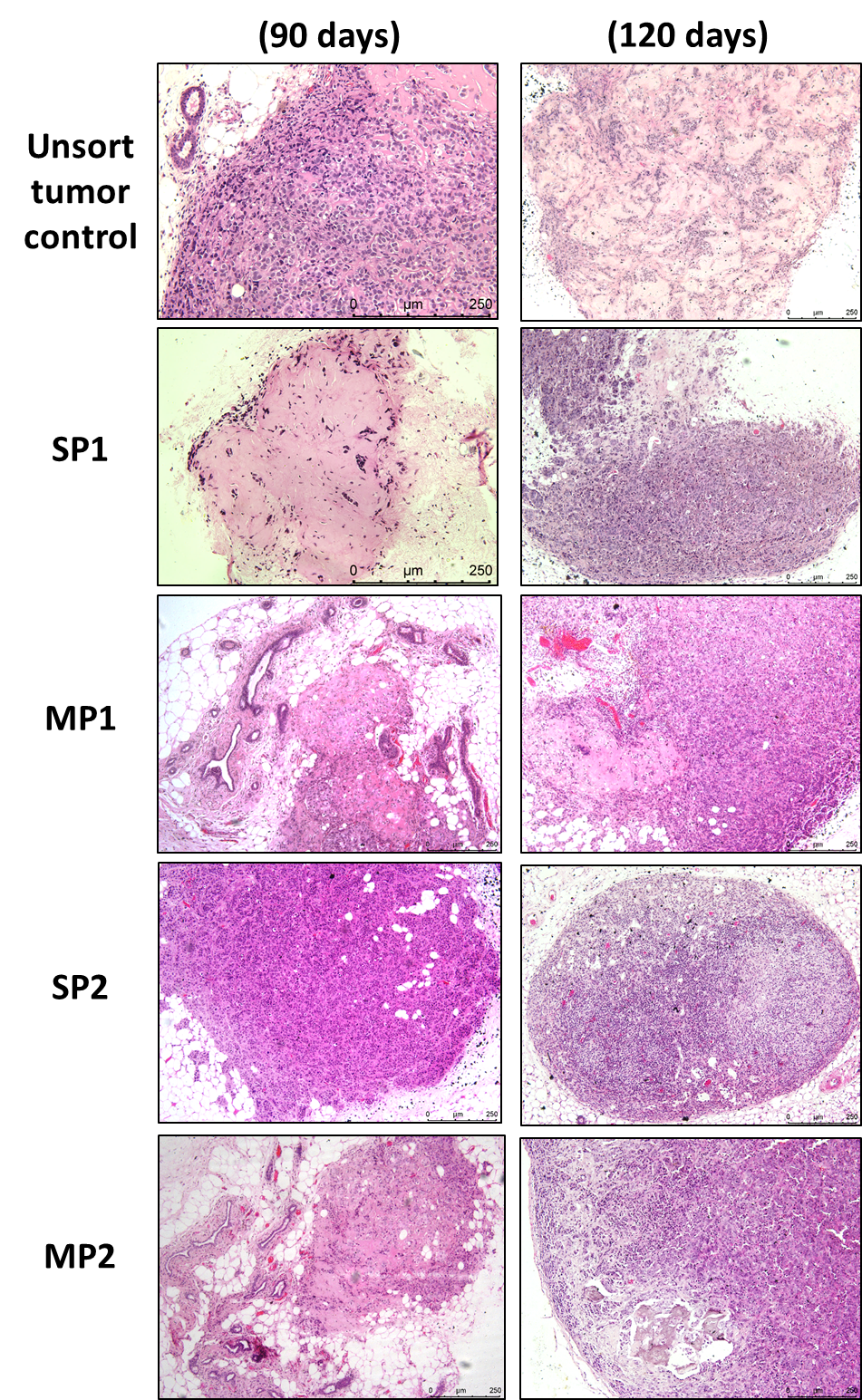

Supplement: Supplementary file 1 [file ijms-24-03497-s001.zip › Figure S2.tif]
